# Unsupervised Hierarchical Classification Approach for Imprecise Data in the Breast Cancer Detection

**DOI:** 10.3390/e24070926

**Published:** 2022-07-03

**Authors:** Mario Fordellone, Paolo Chiodini

**Affiliations:** Medical Statistics Unit, Universitiy of Campania “Luigi Vanvitelli”, 81100 Naples, Italy; paolo.chiodini@unicampania.it

**Keywords:** unsupervised classification, hierarchical classification, interval-valued data, imprecise data, cancer detection, cancer classification

## Abstract

(1) Background: in recent years, a lot of the research of statistical methods focused on the classification problem in presence of imprecise data. A particular case of imprecise data is the interval-valued data. Following this research line, in this work a new hierarchical classification technique for multivariate interval-valued data is suggested for diagnosis of the breast cancer; (2) Methods: an unsupervised hierarchical classification method for imprecise multivariate data (called HC-ID) is performed for diagnosis of breast cancer (i.e., to discriminate between benign or malignant masses) and the results have been compared with the conventional (unsupervised) hierarchical classification approach (HC); (3) Results: the application on real data shows that the HC-ID procedure performs better HC procedure in terms of accuracy (HC-ID = 0.80, HC = 0.66) and sensitivity (HC-ID = 0.61, HC = 0.08). In the results obtained by the usual procedure, there is a high degree of false-negative (i.e., benign cancer diagnosis in malignant status) affected by the high degree of variability (i.e., uncertainty) characterizing the worst data.

## 1. Introduction

Among all types of cancers, breast cancer is one of the leading causes of death among middle-aged and old women. According to the World Health Organization (WHO), after two decades, lung cancer loses its sad record as the most widespread neoplasm. Breast cancer is now the most common oncological pathology. About 2.3 million new cases of breast cancer were diagnosed in 2020: 11.7% of all new cases of cancer [1,2].

Thus, prevention and an early diagnosis of breast tumors are immediate demands from society. Primary prevention is difficult as the causes of the disease are not well understood. However, if it can be detected at its early stage, the success rate of survival is quite high [3]. Physical examination and mammography are the best ways to make an early diagnosis of the disease. A precise detection, however, often depends on the visibility of microcalcifications in the mammogram. It is still challenging for radiologists to differentiate between benign and malignant cases. The existence of breast tumors is usually reflected in the mammogram. Some of the important signs of malignancy are: clustered calcifications, poorly defined masses, isolated dilated ducts, etc. However, all of these are not equally reflected in the mammograms [4].

Doctors physically look at the mammograms to detect deformations that may be taken as an indicator of cancerous changes and this could suffer from the human error and error with a visual inspection, which may further be enhanced by the poor quality of the mammogram images [5]. To try to solve these problems there is a demand for intelligent systems (e.g., statistical approaches, machine learning techniques, mathematical models, etc.) for early detection of tumors, assessment of their malignancy and monitoring of the same on the basis of multivariate features. In this direction, even some aiding tools would be of immense help. The efficiency and effectiveness of this process can be increased if tumors are detected and classified automatically through computers as benign or malignant [6].

In the breast cancer detection field, many classification approaches have been applied for diagnostic purposes, e.g., [6] use rank nearest neighbor (RNN) classification rules, in [7] authors focus on association rules (AR) and artificial neural network (ANN), in [8] authors propose a non-parametric statistical model, in [9] is shown a comparative study of machine learning algorithms, in [10] a novel approach using data mining techniques is presented, and in [11] authors use machine learning approaches as well as Naive Bayes (NB) classifier and k-nearest neighbor (KNN). For a detailed review, the reader can refer to [12]. Notice that all these proposals are based on supervised techniques. In the supervised learning model, the algorithms learn from labeled data (i.e., the structure groups are known). After understanding the data, the algorithm determines which label should be given to new data by associating patterns to the unlabeled new data [13]. In medical and statistical literature, little attention is paid to research works where unsupervised techniques for cancer detection have been proposed. In unsupervised learning, the algorithms segregate the data in a data set in which the data is unlabeled (i.e., the structure groups are unknown) based on some hidden features in the data. This function can be useful for discovering the hidden structure of data and for tasks like anomaly detection [14]. In [15], authors tried to predict the disease based on relevant features in the data through the use of unsupervised algorithms; in [16] authors used the *K*-means (KM) algorithm to evaluate the impact of clustering for the early detection of breast cancer, using centroid initialization, distance measures, and split methods; in [17] authors proposed a review based on several ultrasound image segmentation techniques, mainly focus on eight clustering methods over the last 10 years, and they showed the advantages and disadvantages of these approaches; in [18] authors proposed a comparative study where three different unsupervised learning models have been used for breast cancer detection.

However, in real-life applications, the results of measurements are never precise (i.e., some degree of uncertainty that characterizes them there is). The uncertainty of a measurement can be defined as the interval on the measurement scale within which the true value lies with a specified probability when all sources of error have been taken into account [19,20]. From a statistical point of view, in recent years the research of statistical methodologies to analyze complex structures of data has increased. In particular, a lot of attention has been focused on the imprecise data [21]. For example, the concentration of toxic substances in different environmental media are imprecise quantities and then, their measurements are not precise. In this work, an unsupervised hierarchical classification method for imprecise multivariate data (called HC-ID) is performed for the diagnosis of breast cancer (i.e., to discriminate between benign or malignant masses) and the results have been compared with the conventional (unsupervised) hierarchical classification approach (HC). Notice that both HC approaches are performed by the *complete linkage* [22] method and then, in agglomerative way. For other examples of HC application in breast cancer detection, the reader can refer to [23,24,25]. In recent years, the research of statistical methods to analyze complex structures of data has increased. In particular, a lot of attention has been focused on the unsupervised and supervised classification problem in presence of imprecise data [26,27,28,29]. The simplest case of imprecise data is the interval-valued data [26,30]. In the literature on data analysis, a great deal of attention is paid to statistical methods to treat interval-valued data, in different research areas [26,30,31,32,33,34]. The novelty of this work is to consider the variability (i.e., the uncertainty) of the data in the classification procedure. In many medical research areas, such as in cancer detection studies, the results can be affected by measurement uncertainty, and this, in turn, could affect the statistical analysis reliability. In these cases, researchers should be interested to consider the uncertainty as a crucial part of the information rather than a simple noise factor.

## 2. Materials and Methods

### 2.1. Methodology of the Proposed Approach

We can formalize an interval-valued data as $x_{ij}=\left[{\underline{x}}_{ij},{\bar{x}}_{ij}\right]$, i=1,⋯,n and j=1,⋯,J; where $x_{ij}$ is the *j*-th interval valued variable observed on the *i*-th observation, ${\underline{x}}_{ij}$ and ${\bar{x}}_{ij}$ denote the lower and upper bounds of the interval, respectively, (i.e., the minimum and maximum values registered for the *j*-th interval-valued variable with respect to the *i*-th observation). Then, in an n×J interval-valued data matrix, each observation is represented as a hyperrectangle (in $R^{J}$) having $2^{J}$ vertices. However, a simpler notation of interval-valued data consists to consider centers and radii, separately. In particular, we can indicate C the n×J*centers matrix* (or midpoints matrix) whose generic element $c_{ij}=2^{-1}\left({\underline{x}}_{ij}+{\bar{x}}_{ij}\right)$ is the center (midpoint) of the associated interval. Furthermore, we can define R the n×J*radii matrix* whose generic element $r_{ij}=2^{-1}\left({\bar{x}}_{ij}-{\underline{x}}_{ij}\right)$ is the radius of the associated interval. Then, by considering this reformulation of the interval-valued data, the interval-valued matrix can be formalized as follows:

(1) $$X\equiv \left\{x_{ij}=\left[c_{ij},r_{ij}\right]:i=1,\cdots ,n;\;j=1,\cdots ,J\right\}$$

**Figure 1:**
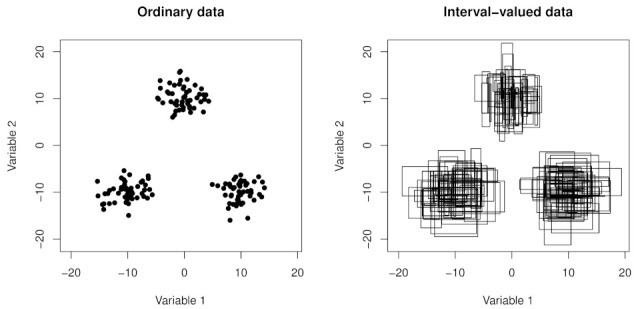
Artificial data generated by three bi-variate Normal distributions. To the left we have dataset in ordinary form; to the right we have interval-valued dataset.

In the left plot of Figure 1 is represented a bi-dimensional dataset in ordinary form (i.e., with a radius equal to zero), while in the right one is represented a bi-dimensional interval-valued dataset.

We can note that a structure of three groups characterizes our datasets. In particular, 300 observations by three different bi-variate normal distributions (i.e., 100 for each group) have been generated in order to obtain the left plots. Subsequently, other three bi-variate normal distributions have been used to obtain random radii for the right plot.

The generic interval-valued data pertaining to the *i*-th observation with respect to the *j*-th interval-valued feature can be shown as the pair ($c_{ij}$,$r_{ij}$), i=1,⋯,n and j=1,⋯,J, where $c_{ij}$ denotes the center and $r_{ij}$ the radius of the interval (i.e., $x_{ij}=c_{ij}\pm r_{ij}$). In the literature, several metrics have been suggested for interval-valued.

Let ${\bar{I}}_{ij}$ be the *i*-th interval with respect to the *j*-th feature, within an interval pair $\left\{{\bar{I}}_{ij},{\bar{I}}_{i^{\prime }j}\right\}$, OR between ${\bar{I}}_{ij}$ and ${\bar{I}}_{i^{\prime }j}$ is defined as

(2) $$OR\left({\bar{I}}_{ij},{\bar{I}}_{i^{\prime }j}\right)=\frac{|{\bar{I}}_{ij}\cap {\bar{I}}_{i^{\prime }j}|}{|{\bar{I}}_{ij}|},$$

where $|{\bar{I}}_{ij}\cap {\bar{I}}_{i^{\prime }j}|$ is the size of the intersection between ${\bar{I}}_{ij}$ and ${\bar{I}}_{i^{\prime }j}$, while $|{\bar{I}}_{ij}|$ is the size of interval ${\bar{I}}_{ij}$. OR for an interval in a given pair will fall under one of the following cases:

- $OR({\bar{I}}_{ij},{\bar{I}}_{i^{\prime }j})=1$, when ${\bar{I}}_{ij}$ and ${\bar{I}}_{i^{\prime }j}$ are identical;
- $OR({\bar{I}}_{ij},{\bar{I}}_{i^{\prime }j})=0$, when ${\bar{I}}_{ij}$ and ${\bar{I}}_{i^{\prime }j}$ are disjointed;
- Otherwise, $0\leq OR({\bar{I}}_{ij},{\bar{I}}_{i^{\prime }j})\leq 1$.

Thus, the overlapping ratio-based similarity measure $S_{OR}$ takes into consideration the reciprocal similarity of intervals within a pair in order to estimate their overall similarity. Formally, $S_{OR}$ for a pair of intervals, ${\bar{I}}_{ij}$ and ${\bar{I}}_{i^{\prime }j}$, is the vectors sum (i.e., the norm) of their overlapping ratios:

(3) $$S_{OR}\left({\bar{I}}_{ij},{\bar{I}}_{i^{\prime }j}\right)=\left|OR\left({\bar{I}}_{ij},{\bar{I}}_{i^{\prime }j}\right),OR\left({\bar{I}}_{i^{\prime }j},{\bar{I}}_{ij}\right)\right|=\left|\frac{|{\bar{I}}_{ij}\cap {\bar{I}}_{i^{\prime }j}|}{|{\bar{I}}_{ij}|},\frac{|{\bar{I}}_{ij}\cap {\bar{I}}_{i^{\prime }j}|}{|{\bar{I}}_{i^{\prime }j}|}\right|.$$

Then, the overlapping ratio-based similarity measure for a pair of intervals ${\bar{I}}_{i}$ and ${\bar{I}}_{i^{\prime }}$ in the *J*-dimensional space, is defined as

(4) $$S_{OR}^{J}\left({\bar{I}}_{i},{\bar{I}}_{i^{\prime }}\right)=\sqrt[{J}]{\sum_{j=1}^{J}{\left[\frac{|{\bar{I}}_{ij}\cap {\bar{I}}_{i^{\prime }j}|}{|{\bar{I}}_{ij}|}+\frac{|{\bar{I}}_{ij}\cap {\bar{I}}_{i^{\prime }j}|}{|{\bar{I}}_{i^{\prime }j}|}\right]}^{2}}.$$

Note that in this work a distance measure $D_{OR}^{J}\left({\bar{I}}_{i},{\bar{I}}_{i^{\prime }}\right)$ [35] has been used, which can easily be derived as

(5) $$D_{OR}^{J}\left({\bar{I}}_{i},{\bar{I}}_{i^{\prime }}\right)=\sqrt[{J}]{\sum_{j=1}^{J}{\left[\left(1-\frac{|{\bar{I}}_{ij}\cap {\bar{I}}_{i^{\prime }j}|}{|{\bar{I}}_{ij}|}\right)+\left(1-\frac{|{\bar{I}}_{ij}\cap {\bar{I}}_{i^{\prime }j}|}{|{\bar{I}}_{i^{\prime }j}|}\right)\right]}^{2}}.$$

The final result is a distance matrix characterized by intervals. In this work, we use this particular distance matrix for the classification purpose of imprecise data. In particular, a hierarchical classification method with an interval-valued distance matrix and a *complete linkage* approach has been performed. This new model is called HC-ID.

### 2.2. Description of the Breast Cancer Example Data

In this work, an analysis of the Breast Cancer Wisconsin (Diagnostic) dataset is performed (https://archive.ics.uci.edu/ml/datasets/breast+cancer+wisconsin+(diagnostic) accessed on 27 June 2022). This data set was created by [36] and it has been very used for training statistical methods (e.g., [37]). To create the dataset Dr. Wolberg used fluid samples, taken from patients with solid breast masses and an easy-to-use graphical computer program called Xcyt, which is capable of performing the analysis of cytological features based on a digital scan. The program uses a curve-fitting algorithm, to compute ten features from each one of the cells in the sample, then it calculates the mean value, extreme value and standard error of each feature for the image, returning a 30 real-valued vector. Dataset consists of 569 patients, 357 with benign diagnosis and 212 with malignant status.

Attribute Information (response variable):

- Diagnosis (M = malignant, B = benign).

Ten real-valued features are computed for each cell nucleus:

1. Radius (mean of distances from center to points on the perimeter);
2. Texture (standard deviation of gray-scale values);
3. Perimeter;
4. Area;
5. Smoothness (local variation in radius lengths);
6. Compactness (perimeter${}^{2}$/area − 1.0);
7. Concavity (severity of concave portions of the contour);
8. Concave points (number of concave portions of the contour);
9. Symmetry;
10. Fractal dimension (“coastline approximation” − 1).

The mean, standard error and “worst” or largest (mean of the three largest values) of these features were computed for each image, resulting in 30 features. For instance, field 3 is the mean radius, field 13 is radius se, and field 23 is the worst radius. For breast cancer diagnosis we have compared the results obtained by the proposed HC-ID approach with those one obtained by the conventional HC approach. In particular, to obtain the HC-ID model the dissimilarity measure for interval-valued data based on the overlapping ratio (OR) proposed by [35] has been applied to the interval-valued dataset. Then, we have used the 10 *worst* features (i.e., the feature with greater uncertainty/variability) as the centers of the interval data, while the *standard deviations* (i.e., the degree of uncertainty) are the radii. In this way, the classification procedure includes also the degree of uncertainty (i.e., variability) characterizing data and more homogeneous and separated groups are guaranteed (for details on the imprecise data concept, the reader can refer to [26,28,38]). Notice that for the classification we assume that the observed diagnosis groups is unknown (i.e., unsupervised classification).

### 2.3. Statistical Analysis

For the hierarchical classification model the hclust R package was used, while to obtain the interval-valued distance matrix based on $D_{OR}^{J}\left({\bar{I}}_{i},{\bar{I}}_{i^{\prime }}\right)$, the reader can refer to web page: https://github.com/mfordellone/Unsupervised-hierarchical-classification-approach-for-imprecise-data-in-the-breast-cancer-detection.git accessed on 27 June 2022. Notice that HC-ID is an unsupervised technique and then, it helps the analyst to identify data-driven patterns that may warrant further investigation but the prediction is not provided. You can easily use HC-ID to perform clustering, and from there for every new data point, you just find which cluster it matches most closely.

To evaluate the diagnostic performance of the HC-ID model sensitivity, specificity, positive predictive value, negative predictive value, positive likelihood ratio (LR+), negative likelihood ratio (LR-), and the accuracy rate have been used. 

## 3. Results

**Figure 2:**
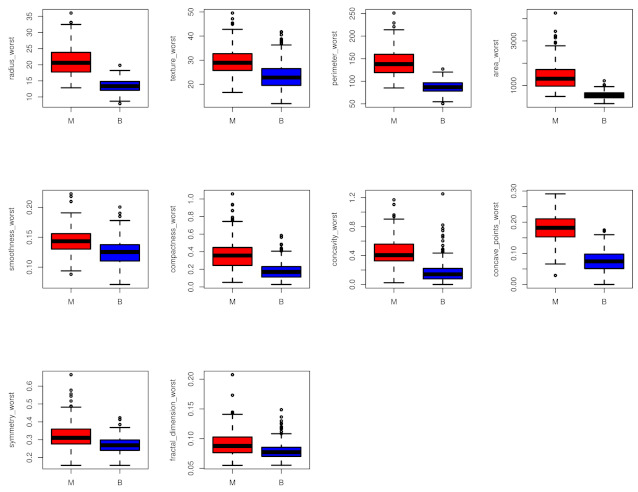
Variables distribution with respect to the observed diagnosis groups of data.

The dataset consists of 569 patients, where 212 (37.26%) have malignant breast cancer and 357 (62.74%) have benign. Figure 2 shows all the variables distribution (*worst*) included in the analysis with respect to the observed diagnosis groups of data (M: Malignant, B: Benign).

**Figure 3:**
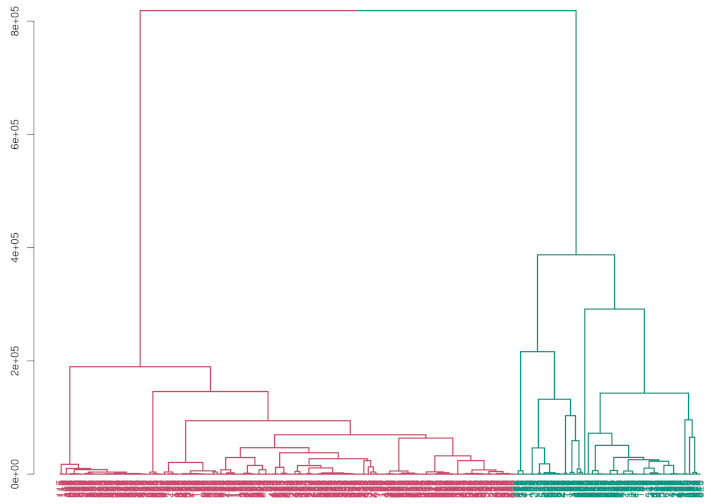
Dendrogram obtained by HC applied on interval-valued data.

**Figure 4:**
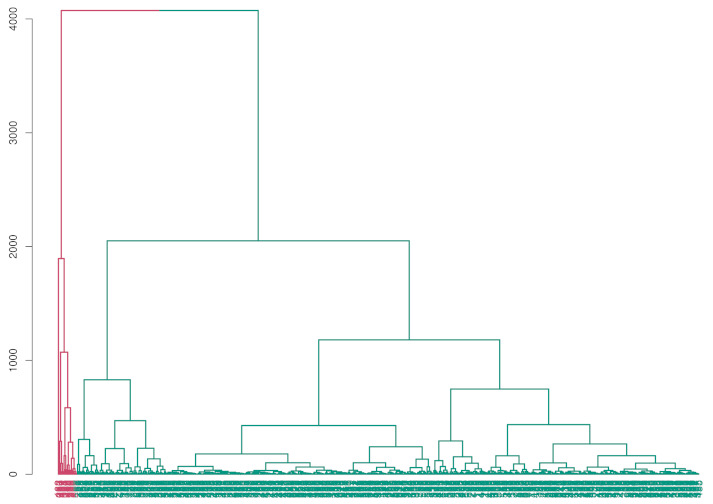
Dendrogram obtained by HC applied on *worst* data.

By applying HC-ID (i.e., the use of the interval-valued distance matrix obtained via OR approach) the automatic classification shown by the dendrogram in Figure 3 is obtained. The predicted diagnosis group proportions are 28.82% for malignant breast cancer and 71.18% for benign breast cancer. By applying the conventional HC (i.e., the use of data points to obtain the distance matrix of the *worst* data), the automatic classification shown by dendrogram in Figure 4 is obtained. In this case, the predicted diagnosis group proportions are 2.98% for malignant breast cancer and 97.02% for benign breast cancer.

The results show that the classification procedure based on interval-valued data performs better than the usual procedure in terms of accuracy and sensitivity. In the results obtained by the usual procedure, there is a high degree of false-negative (i.e., benign cancer diagnosis in malignant status) affected by the high degree of variability (i.e., uncertainty) characterizing the *worst* data. Additionally, the dendrograms represented in Figure 3 and Figure 4 show a greater homogeneity in the partition obtained by the interval-valued approach. Finally, Table 1 shows the summary of the performance obtained by the two approaches.

In columns 1 and 4 of the table are shown the estimated values of diagnostic evaluation measures obtained by HC-ID and HC approaches, respectively; in columns 2–3 and 5–6 are shown the estimated confidence intervals at 95% (i.e., Lower 95% and Upper 95% are the lower limits and the upper limit of the confidence interval) on the diagnostic evaluation measures obtained by HC-ID and HC approaches, respectively.

## 4. Discussion

In this work, an unsupervised hierarchical classification method for interval-valued multivariate data (HC-ID) is performed for diagnosis of the breast cancer. In particular, a methodology able to discriminate between benign or malignant breast masses has been proposed. Moreover, in order to show the good performance of the proposed classification model comparison with the conventional (unsupervised) hierarchical classification approach is carried out.

The principal novelty of the proposed approach is the use of an unsupervised classification approach. In fact, the most important previous proposals ([9,10,11], etc.) are based on the use of classification methodologies where the observed diagnosis groups are known. However, in some real cases, this information could not be available.

For application purpose, an analysis of the Breast Cancer Wisconsin (Diagnostic) dataset (https://archive.ics.uci.edu/ml/datasets/breast+cancer+wisconsin+(diagnostic) accessed on 27 June 2022) created by [36] is performed. In particular, we have used the 10 *worst* features as the centers of the interval data, while the *standard deviations* are the radii. In this way, the classification procedure includes also the degree of uncertainty (i.e., variability) characterizing data and more homogeneous and separated groups are guaranteed. In fact, the results show that our proposal performs better than the conventional procedure (i.e., the HC approach) in terms of accuracy, sensitivity and negative predictive value. However, the specificity and the positive predictive value obtained by the usual procedure are equal to 1 but, unfortunately, also the false-negative rate increases. Moreover, the LR+ obtained by HC is characterized by high variability and shows a very large confidence interval. We think that this result is affected by the high degree of variability (i.e., uncertainty) characterizing the *worst* data. Moreover, we think that the high rate of false-negative in cancer detection fields is a serious problem. In particular, false-negative tests at diagnosis of early disease and of relapse resulted in diagnostic and therapeutic delays.

We think that the principal advantages of the HC-ID approach consist of (i) to include the uncertainty of the data in the classification procedure that leads to more homogeneous partitions of subjects; (ii) the possibility to consider a multi-group approach that encourages the use of the procedure for different purposes (e.g., stages detection or identification of prognosis classes); (iii) the external procedure of uncertainty estimation that leads to fix a different kind of measures (e.g., IQR, specific percentile differences, other intervals symmetrical or not symmetrical with respect the point data, etc.).

Whereas, the principal disadvantages consist of (i) the correct estimation of uncertainty since it is not simple; (ii) to fix a constant uncertainty measure of the subjects is a very strong assumption. The subjects could have some characteristics to affect the variability degree in different measures; (iii) the approach is not very adequate in cases with small sample sizes. In these cases, the radii of the imprecise datum could be very high and the risk to associate the biggest weight to the uncertainty than the point data is hard to handle. However, in Appendix A a validation study is proposed in order to study the HC-ID model behavior for different sample size.

Finally, we think that our proposed approach is very useful for cancer diagnostic purposes in the cases where there is a marked variability in the subjects’ features and where the outcome information is incomplete or not available. For future research, could be interesting to provide a validation of the results using other datasets and other cancer types, because at moment this is a real limit of this work. Additionally, the comparison with other classification statistical models could be an interesting development for this research line.

## Figures and Tables

**Table 1:** Comparison of the performances obtained by interval-valued approach and conventional approach.

	Interval-Valued Approach			Conventional Approach		
	Estimate	Lower 95%	Upper 95%	Estimate	Lower 95%	Upper 95%
Sensitivity	0.613	0.544	0.679	0.080	0.047	0.125
Specificity	0.905	0.869	0.933	1.000	0.990	1.000
Pos.Pred.Val.	0.793	0.723	0.852	1.000	0.805	1.000
Neg.Pred.Val.	0.798	0.755	0.836	0.647	0.605	0.687
LR+	6.439	4.596	9.020	58.826	3.556	973.204
LR−	0.427	0.360	0.508	0.920	0.883	0.957
Accuracy	0.796	0.761	0.829	0.657	0.617	0.696

## Data Availability

Not applicable.

## Abbreviations

The following abbreviations are used in this manuscript:

MDPI	Multidisciplinary Digital Publishing Institute
DOAJ	Directory of open access journals
TLA	Three letter acronym
LD	Linear dichroism

## Appendix A. Validation Study

In order to study the HC-ID model behavior for different sample size, the model has been applied on two sub-samples of 150 and 300 subjects random selected, without replacement, from the Breast Cancer Wisconsin dataset. Then, in the first case dataset consists of 150 patients, where 55 (36.67%) have a malignant breast cancer and 95 (63.33%) have the one benign; in the second case dataset consists of 300 patients, where 114 (37.26%) have a malignant breast cancer and 186 (62.74%) have the one benign. Table A1 and Table A2 show the results obtained by HC-ID model compared with those one obtained by the conventional HC model.

**Table A1:** Comparison of the performances obtained by interval-valued approach and conventional approach on a randomized sub-sample of 150 subjects.

	Interval-Valued Approach			Conventional Approach		
	Estimate	Lower 95%	Upper 95%	Estimate	Lower 95%	Upper 95%
Sensitivity	1.000	0.962	1.000	0.036	0.004	0.125
Specificity	0.709	0.571	0.824	1.000	0.962	1.000
Pos.Pred.Val.	0.856	0.776	0.915	1.000	0.158	1.000
Neg.Pred.Val.	1.000	0.910	1.000	0.642	0.559	0.719
LR+	3.438	2.275	5.193	8.571	0.419	175.363
LR−	0.007	0.000	0.118	0.964	0.915	1.014
Accuracy	0.893	0.833	0.938	0.647	0.565	0.723

**Table A2:** Comparison of the performances obtained by interval-valued approach and conventional approach on a randomized sub-sample of 300 subjects.

	Interval-Valued Approach			Conventional Approach		
	Estimate	Lower 95%	Upper 95%	Estimate	Lower 95%	Upper 95%
Sensitivity	0.935	0.890	0.966	0.009	0.000	0.048
Specificity	0.623	0.527	0.712	1.000	0.980	1.000
Pos.Pred.Val.	0.802	0.743	0.853	1.000	0.025	1.000
Neg.Pred.Val.	0.855	0.761	0.923	0.622	0.564	0.677
LR+	2.480	1.953	3.149	4.878	0.200	118.743
LR−	0.104	0.059	0.182	0.991	0.974	1.008
Accuracy	0.817	0.768	0.859	0.623	0.566	0.678

The two tables show that HC-ID performs better than the conventional HC model except in specificity and positive predictive value. In particular, we can see that the results are not affected by the sample size since they are very similar than the results reported in Table 1.
